# Mimicking Metastasis: Endobronchial Tuberculosis Presenting as a Mass in a Pediatric Patient With Yolk Sac Tumor

**DOI:** 10.7759/cureus.65277

**Published:** 2024-07-24

**Authors:** Souvik Sarkar, Hari Kishan Gonuguntla, Preeti Vidyasagar

**Affiliations:** 1 Respiratory Medicine, Datta Meghe Institue of Higher Education and Research, Wardha, IND; 2 Intervention Pulmonology, Yashoda Hospital, Hyderabad, IND

**Keywords:** testicular germ cell tumors, bronchoscopic biopsy, yolk sac tumor, endobronchial tuberculosis, endobronchial metastasis

## Abstract

Before the advent of an effective antitubercular treatment for tuberculosis and bronchoscopy, endobronchial tuberculosis (EBTB) often greatly contributed to airway stenosis and lung atelectasis in children. Even after the advent of efficacious therapy, EBTB poses major challenges for pediatric patients, manifesting as airway stenosis or obstruction. We report a case of a two-year-old male with a previous history of yolk sac testicular tumor whose follow-up PET-CT scan showed right middle lobe collapse. Flexible bronchoscopy demonstrated endobronchial mass, and biopsy revealed EBTB, excluding metastasis. This case illustrates varied presentations of tuberculosis and highlights the significance of early diagnosis with bronchoscopy in treating this condition before it can lead to severe complications. Antitubercular therapy must be initiated at the earliest when managing EBTB. The follow-up procedures must be diligent, and timely interventions should be made for optimal patient outcomes despite the availability of improved diagnostic techniques and treatment methods.

## Introduction

Before the era of effective antitubercular therapy and bronchoscopy, endobronchial tuberculosis (EBTB) was a severe healthcare issue, often leading to airway stenosis and atelectatic lung [1]. EBTB frequently affects children, with around 40% of total cases of pulmonary tuberculosis reported in the pediatric population. EBTB frequently leads to complications such as airway stenosis or obstruction, resulting in a hampered quality of life and may sometimes even be fatal [2]. With the increased use of diagnostic bronchoscopy [3] and effective antitubercular treatment, the occurrence of complications of EBTB has significantly declined. However, it remains a challenging situation for pediatric patients.

In this report, we present an interesting case of a two-year-old male, a known and operated case of yolk sac testicular tumor, who presented with a collapse of the right middle lobe of the lung observed in a follow-up PET-CT scan. Flexible bronchoscopy helped in the visualization of an endobronchial mass, and a forceps biopsy of the same ruled out any metastasis from the previous disease, confirming the diagnosis of EBTB. To prevent the grave complications of EBTB, a close follow-up and, if necessary, an intervention might be essential, specifically in the tumorous variant of EBTB [4].

## Case presentation

A two-year-old male, a known case of yolk sac testicular tumor, presented to the pediatric outpatient department for a regular follow-up. He had undergone a right orchidectomy a year ago and had received one cycle of chemotherapy with PEB (cisplatin, etoposide, and bleomycin) postoperatively. The child was now asymptomatic, and a thorough clinical examination was also done, which showed normal vitals, normal height and weight for age, and no palpable lymph nodes. He was then advised to get a PET-CT to check for any metastatic foci or local site recurrence.

**Figure 1:**
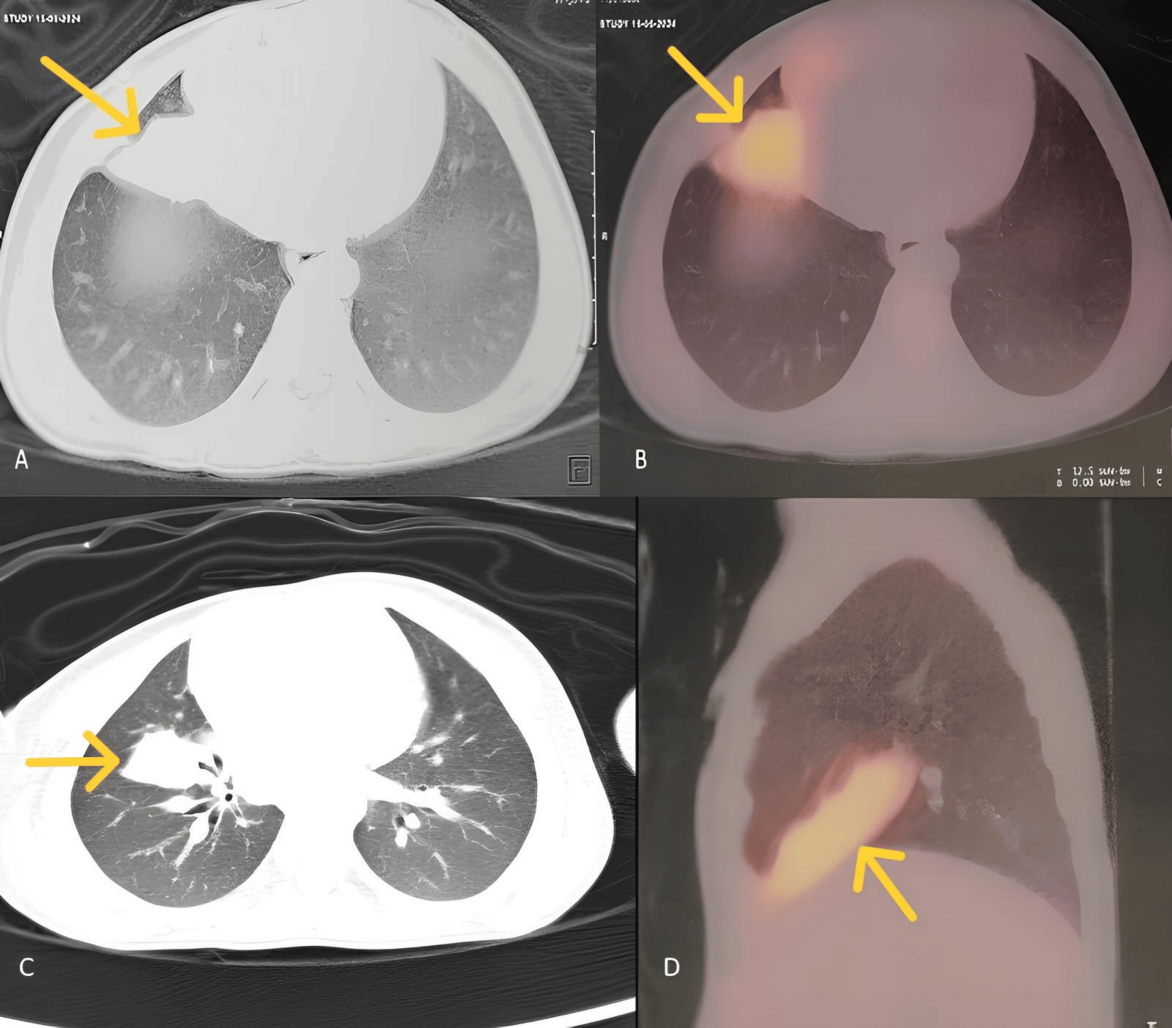
Imaging results (A) Axial view of contrast-enhanced CT of thorax showing an enhancing interfissural mass (yellow arrow) in the middle lobe of the right lung (size 1.3 x 2.1 x 3.7 cm). (B) A FDG-avid lesion with a maximum standard uptake value (SUV max) of 8.0 (yellow arrow). (C) Interfissural consolidation with another possibility of mass, in the right middle lobe (yellow arrow). (D) Saggital section view showing oblong mass-like lesion in right middle lobe (yellow arrow), with FDG uptake CT: computed tomography; FDG: fluoro-deoxy-glucose

Surprisingly, the PET-CT showed fluoro-deoxy-glucose (FDG)-avid heterogeneously enhancing interfissural oblong consolidatory mass in the right middle lobe of the right lung measuring 1.3 x 2.1 x 3.7 cm with a maximum standard uptake value (SUV max) of 8.0. There were also mild FDG-avid uptakes in the right paratracheal and subcarinal lymph nodes, the largest measuring 0.7 x 0.7 cm with an SUV max of 4.0. The rest of the scan showed no definite evidence of any metabolically active foci/enhancing lesion in the postoperative site of the right scrotum to suggest residual or recurrent malignant disease (Figure 1).

**Figure 2:**
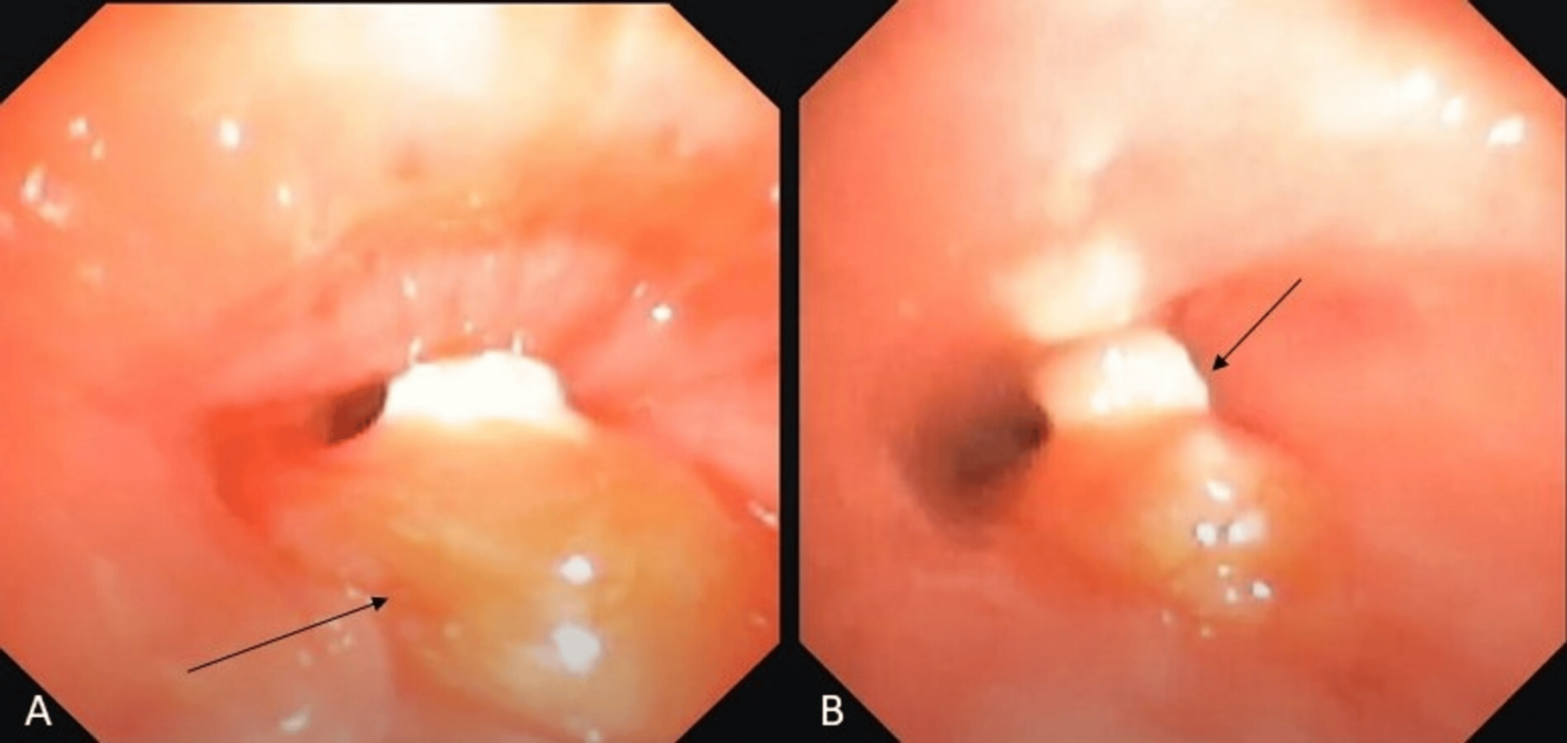
Bronchoscopy results (A) Bronchoscopic images of the endobronchial growth occluding more than 70% of the lumen arising from the postero-lateral wall of bronchus intermedius (black arrow). (B) Pus/caseous material seen at the tip of the mass (black arrow)

The patient's serum alpha-fetoprotein levels were 0.804 ng/mL (normal range 5-10 ng/mL), and his beta-HCG level was less than 2.39 (normal); the rest of the routine blood investigations revealed no significant abnormality. He was planned to be taken up for flexible bronchoscopy-guided biopsy from the lung lesion with a thin bronchoscope of 3.0 mm outer diameter under general anesthesia. The bronchoscopy showed an endobronchial mass with a caseous material at the tip of the lesion arising from the postero-lateral wall of the right intermediate bronchus abutting the lumen of the middle lobe bronchus, leading to the collapse of the middle lobe; also, retained thick secretions were seen oozing out from the middle lobe bronchus (Figure 2).

**Figure 3:**
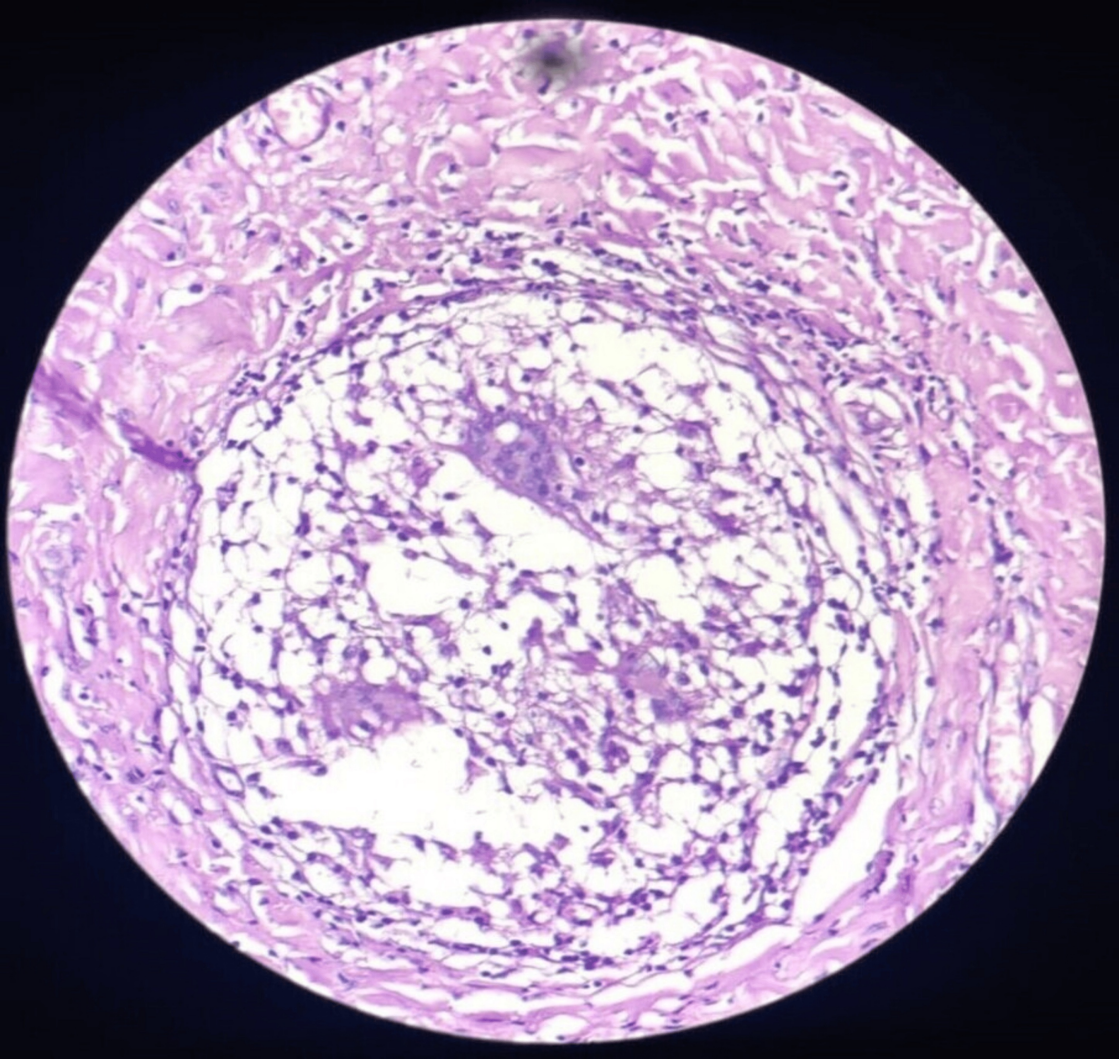
Biopsy sample Hematoxylin & eosin, 40x section shows central necrotic zone surrounded by epithelioid histiocytes with a varied number of multinucleated giant cells and lymphocytes. Histopathological features are suggestive of necrotizing granuloma

A forceps biopsy was taken from the mass and sent for histopathological examination and bronchoalveolar lavage was also taken from the right lung all segments and was sent for Ziehl-Neelson staining and Cartridge-Based Nucleic Acid Amplification Test (CBNAAT). The AFB staining and CBNAAT turned out to be negative, but the biopsy sample of the endobronchial growth showed aggregates of epithelioid histocytes, giant cells, mononuclear inflammation, and necrosis, suggesting necrotizing granulomatous inflammation, likely to be tuberculosis. The biopsy sample did not show any evidence of malignancy or metastasis (Figure 3).

The patient was started on first-line antitubercular treatment (Isoniazid, rifampicin, pyrazinamide, and ethambutol) and was advised to follow up at two months to undergo a bronchoscopy.

## Discussion

According to Jung et al., EBTB can affect any part of the tracheobronchial tree, with the primary bronchi, superior lobar bronchi, and right middle lobar bronchus being the most commonly affected sites. The researchers classified EBTB into three types: single-level, multiple-level, and central EBTB. Single-level EBTB involves only one part of the trachea, main bronchus, or lobar bronchus [1-3]. Multiple-level EBTB affects two or more bronchial levels [2]. Central EBTB, occurring proximal to the lobar bronchi, has the highest potential for developing symptomatic stenosis [3].

As per a study by Jiao et al., patients under five years are at higher risk of developing EBTB as a complication of primary tuberculosis with multiple endobronchial lesions, most frequently presenting as the tumorous type, that too in the right bronchial system, as in our case. In suspected patients, flexible bronchoscopy should be performed at the earliest to detect such endobronchial lesions [2]. The complications related to EBTB include bronchial stenosis or stricture formation, severe airway obstruction or respiratory failure when central airways are involved, post-obstructive bronchiectasis with frequent pneumonia and hemoptysis, and persistent obstructive airway disease [1,4,5].

More than 90% of testicular cancers arise from germ cells and are divided into seminomas (SGCTs) and non-seminomas (NSGCTs). NSGCTs mainly include embryonal carcinoma, yolk sac carcinoma, choriocarcinoma, and teratoma [6]. Although the chest commonly hosts metastases from testicular tumors, only seven endobronchial metastasis (EBM) cases have been reported till now, making it an extremely rare phenomenon [7]. Chung et al. have provided a classification of the subtypes of EBTB based on bronchoscopic features (Table 1) [8].

**Table 1:** Bronchoscopic classification of endobronchial tuberculosis and their incidence

Subtype	Bronchoscopic findings
Actively caseating (43%)	Swollen hyperemic bronchial mucosa covered with whitish cheese-like material
Edematous-hyperemic (14%)	Extensive mucosal swelling with surrounding hyperemia
Fibrostenotic (10.5%)	Marked narrowing of the bronchial lumen with fibrosis
Tumorous (10.5%)	Endobronchial mass with the surface covered by caseous material, nearly totally occluding the lumen
Granular (11.4%)	Appears like scattered grains of boiled rice
Ulcerative (7.9%)	Ulcerated bronchial mucosa
Nonspecific bronchitis (2.7%)	Mild mucosal swelling and/or hyperemia

The tumorous subtype of EBTB is often misdiagnosed as an endobronchial malignancy due to similar bronchoscopic and radiologic features [8]. In a study by Chung et al., the prognosis of this subtype was found to be the most unpredictable, with complications such as complete bronchial lumen obstruction, fibrostenosis, recurrence of the mass, and the emergence of new tumorous lesions in different bronchi​ [9].

Current management trends primarily involve the administration of antitubercular treatment. The use of steroids remains controversial but can be considered to prevent bronchial stenosis [4]. Bronchoscopic management is crucial for patients who develop complications and can be an alternative option to surgery. Bronchoscopic techniques, such as balloon dilatations, ablation, argon plasma coagulation (APC), cryotherapy, laser therapy, and bronchial stenting, may be used either alone or in combination, depending on the patient's clinical condition [10-13].

## Conclusions

Given our patient’s history of a yolk sac tumor in the right testis, our primary differential diagnosis was endobronchial metastasis. The patient was asymptomatic, with findings observed only on PET-CT. This report highlights the widespread prevalence and varied presentation of tuberculosis and underscores the importance of bronchoscopy in early diagnosis and prompt antitubercular treatment to prevent serious EBTB complications. We recommend performing bronchoscopy in all children presenting with a lobar lung collapse on radiologic studies or with a clinical picture of an endobronchial mass.

## References

[1] Shahzad T, Irfan M (2016). Endobronchial tuberculosis-a review. J Thorac Dis.

[2] Jiao AX, Sun L, Liu F (2017). Characteristics and clinical role of bronchoscopy in diagnosis of childhood endobronchial tuberculosis. World J Pediatr.

[3] Jung SS, Park HS, Kim JO, Kim SY (2015). Incidence and clinical predictors of endobronchial tuberculosis in patients with pulmonary tuberculosis. Respirology.

[4] Kashyap S, Solanki A (2014). Challenges in endobronchial tuberculosis: from diagnosis to management. Pulm Med.

[5] Aggarwal AN, Gupta D, Joshi K, Behera D, Jindal SK (1999). Endobronchial involvement in tuberculosis: a report of 24 cases diagnosed by flexible bronchoscopy. J Bronchology Interv Pulmonol.

[6] Diab AR, Ibrahim I (2022). Endobronchial metastasis of yolk sac testicular tumor. Chest.

[7] Teng CK, Cheng WC, Chen CL, Chen TH, Lin YS, Tu CY (2020). Endobronchial metastases from a primary embryonal carcinoma. Respirol Case Rep.

[8] Chung HS, Lee JH, Han SK, Shim YS, Kim KY, Han YC, Kim WS (1991). Classification of endobronchial tuberculosis by the bronchoscopic fractures. Tuberc Respir Dis.

[9] Chung HS, Lee JH (2000). Bronchoscopic assessment of the evolution of endobronchial tuberculosis. Chest.

[10] Mathur PN, Wolf KM, Busk MF, Briete WM, Datzman M (1996). Fiberoptic bronchoscopic cryotherapy in the management of tracheobronchial obstruction. Chest.

[11] Petrou M, Kaplan D, Goldstraw P (1993). Bronchoscopic diathermy resection and stent insertion: a cost effective treatment for tracheobronchial obstruction. Thorax.

[12] Ball JB, Delaney JC, Evans CC, Donnelly RJ, Hind CR (1991). Endoscopic bougie and balloon dilatation of multiple bronchial stenoses: 10 year follow up. Thorax.

[13] Dumon JF (1990). A dedicated tracheobronchial stent. Chest.

